# A new comparator account of auditory verbal hallucinations: how motor prediction can plausibly contribute to the sense of agency for inner speech

**DOI:** 10.3389/fnhum.2014.00675

**Published:** 2014-08-28

**Authors:** Lauren Swiney, Paulo Sousa

**Affiliations:** ^1^School of Anthropology, Institute of Cognitive and Evolutionary Anthropology, University of OxfordOxford, UK; ^2^Department of History and Anthropology, Institute of Cognition and Culture, Queen’s University BelfastBelfast, UK

**Keywords:** sense of agency, inner speech, comparator model, schizophrenia, efference copy, auditory verbal hallucination

## Abstract

The comparator account holds that processes of motor prediction contribute to the sense of agency by attenuating incoming sensory information and that disruptions to this process contribute to misattributions of agency in schizophrenia. Over the last 25 years this simple and powerful model has gained widespread support not only as it relates to bodily actions but also as an account of misattributions of agency for inner speech, potentially explaining the etiology of auditory verbal hallucination (AVH). In this paper we provide a detailed analysis of the traditional comparator account for inner speech, pointing out serious problems with the specification of inner speech on which it is based and highlighting inconsistencies in the interpretation of the electrophysiological evidence commonly cited in its favor. In light of these analyses we propose a new comparator account of misattributed inner speech. The new account follows leading models of motor imagery in proposing that inner speech is not attenuated by motor prediction, but rather derived directly from it. We describe how failures of motor prediction would therefore directly affect the phenomenology of inner speech and trigger a mismatch in the comparison between motor prediction and motor intention, contributing to abnormal feelings of agency. We argue that the new account fits with the emerging phenomenological evidence that AVHs are both distinct from ordinary inner speech and heterogeneous. Finally, we explore the possibility that the new comparator account may extend to explain disruptions across a range of imagistic modalities, and outline avenues for future research.

## Introduction

Patients seeking psychiatric help often describe unusual experiences and beliefs, such as reporting that their body is under the control of another agent, that they hear voices when there is no one there, or that thoughts are being inserted into their minds. Within psychiatry these reports are classified as delusions of alien control, auditory verbal hallucination (AVH) and delusions of thought insertion, respectively (*Diagnostic and Statistical Manual of Mental Disorders*; 5th ed.; *DSM-V, American Psychiatric Association, 2013*). Such symptoms provide significant weight towards a diagnosis of schizophrenia. While diagnostically distinct, it has been argued that these particular symptoms may share an etiological core, stemming from disruptions to the sense of agency, where the sense of agency refers to the experience of the self as causing and directing one’s actions (e.g., Stephens and Graham, 2003; Jones and Fernyhough, 2007; Langland-Hassan, 2008; Synofzik et al., 2008a; Frith, 2012; Sousa and Swiney, 2013).

Over the last 25 years the comparator account has emerged as the dominant model of the sense of agency and its disruptions in schizophrenia. It draws on a well-established model of the motor control system that holds that the likely sensory consequences of a given motor act are predicted by a forward model, and that this prediction attenuates the actual incoming sensory information. The core idea of the comparator account is that a match between this prediction and the actual sensory information ordinarily gives rise to sense of self-agency. In schizophrenia, disruptions in the process of prediction are proposed to lead to a mismatch, giving rise to a sense of non-self agency (Frith et al., 2000a; Frith, 2005a,b, 2012).

The comparator account of the sense of agency most straightforwardly describes how bodily actions may come to be experienced as non-self produced, giving rise to reports of delusions of alien control (Frith, 2005a). From its inception, however, theorists have held out the possibility that the account could extend to mental acts such inner speech, potentially explaining symptoms of AVH and/or thought insertion (Feinberg, 1978; Frith, 1992, 2005b, 2012). This extension is based on the proposal that inner speech production may draw on the same mechanisms of motor control as bodily actions, and may therefore be subject to the same disruptions in motor prediction. Other theorists have recently taken up the task of providing a precise specification of how such disruptions might manifest relation to inner speech, giving rise to AVH (Seal et al., 2004; Jones and Fernyhough, 2007) or both AVH and thought insertion (Langland-Hassan, 2008).

The basic plausibility of extending the comparator account beyond bodily actions to explain misattributed inner speech is ubiquitously accepted both within the expanding literature on the sense of agency (e.g., Vosgerau and Newen, 2007; Synofzik et al., 2008a) and beyond (e.g., Carruthers, 2011; Whitford et al., 2012). Authors regularly appeal to the account as a plausible explanation for results from behavioral studies (e.g., Li et al., 2002; Johns et al., 2006). The account also forms the basis of a large-scale research program investigating the electrophysiological characteristics of the brain during speech and inner speech in schizophrenia (for a recent review see Ford and Mathalon, 2012). Even among those who critique the account on phenomenological grounds (Wu, 2012) or who argue that the account requires extensions (Synofzik et al., 2008a), the viability of the basic tenants of the comparator account—that inner speech is normally predicted and attenuated and that failures in this process contribute to misattribution—appears to be unproblematically accepted.

Counter to this consensus, we will argue that there are fundamental problems with the comparator account of misattributed inner speech as it has traditionally been formulated. These problems relate both to the plausibility of the account’s specification of inner speech within the motor control system, and to the electrophysiological evidence widely taken to support the account. However, given the emerging evidence for the comparator account as it applies to misattributions of bodily actions in schizophrenia (for a recent overview see Frith, 2012), we acknowledge that the possibility of a unified account of symptoms such as delusions of alien control, AVH and thought insertion provides significant motivation to pursue a comparator account of misattributed inner speech. To this end, we outline a substantially new and revised account of how failures in motor production could give rise to misattributed inner speech. Our account is based on a plausible and cognitively justified model of the production of inner speech in the motor control system, and makes novel predictions about both the phenomenology and neural mechanisms associated with misattributed inner speech.

## The traditional comparator account of misattributed inner speech

### The comparator model of the motor control system

**Figure 1:**
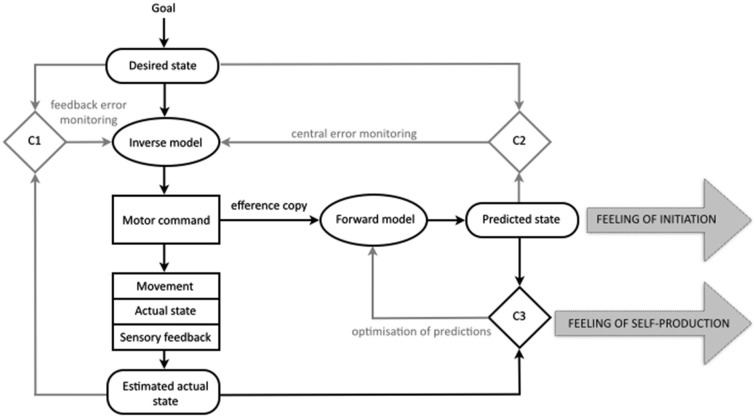
**The comparator model of the motor control system and the comparator account of the sense of agency for bodily action in normal cognition**. Those processes central to the present discussion are in black, the remainder in gray. In this simple model of the motor control system three states are represented internally: the desired state, the predicted state, and the estimated current state. According to the comparator account of the sense of agency for bodily action, the production of the predicted state results in a *feeling of initiation*. Because the predicted state matches the sensory feedback from the new actual state, the comparison in comparator three, gives rise to a *feeling of self-production*. It is proposed that, in schizophrenia, disruptions to the predicted state lead to abnormalities in both the feeling of initiation and the feeling of self-production for bodily actions (for further details see main text; figure modified after Frith et al., 2000b; Blakemore et al., 2002; Synofzik et al., 2008a).

Drawing from ideas on the importance of internal processes of comparison for regulation and control (Helmholtz, 1886; Holst and Mittelstadt, 1950; Sperry, 1950), experimental and computational work over the last years has contributed to our knowledge of the mechanisms constituting motor cognition—those that, at a subpersonal level, generate, control and monitor our physical movements (e.g., Kawato and Wolpert, 1998; Wolpert and Flanagan, 2001; Blakemore et al., 2002; Lindner et al., 2005; Wolpert et al., 2011). The result is the comparator model of motor control. The model posits a system that utilizes feedback and feedforward control loops in conjunction with three comparator mechanisms to direct, control and adjust motor actions. The fundamental job of the motor system is to generate movement by issuing motor commands (see Figure 1). If you want to lift your hand from your lap the motor control system generates the motor command that will guide your hand from your *actual state* (hand in the lap) to your *desired state* (hand above the lap). The representation of your actual state is derived from the current sensory experience of having your hand in your lap, and is therefore always an estimation. The representation of your desired state is based on your goal (to have the hand above the lap). These two representations (estimated actual state, desired state) are compared in the first comparator (C1) and sent to an inverse model that will specify the motor command necessary to get from the estimated actual state (hand in the lap) to the desired state (hand above the lap).

The system also uses the motor command to predict the sensory consequences of a given act; representing the *predicted state* (Miall et al., 1993; Wolpert and Ghahramani, 2000). This is achieved through the production of a duplicate of the motor command known as the efference copy. This efference copy is sent to a forward model that uses it to predict the sensory consequences of issuing the motor command (Holst and Mittelstadt, 1950). This predicted state is compared to the desired state in the second comparator (C2), allowing the motor command to be checked even before it is issued.

The predicted state is also compared to the incoming sensory information (the new actual state, relating to the hand now being above the lap) in the third comparator (C3), allowing for adjustments when the movement does not go according to plan. This comparison in the third comparator is often described in terms of a process of attenuation; the idea is that sensory information that is the result of self-generated movement is attenuated or “canceled out” by the matching predicted state. Evidence for this process includes our inability to tickle ourselves. The incoming sensory information (the tickle) is predicted by the motor control system and so is cancelled out or attenuated (Blakemore et al., 1998). This attenuation is also held to account for other aspects of our phenomenology, such as our experience of a stable visual field (Langland-Hassan, 2008). If you were to move one of your eyes indirectly, without issuing the relevant motor command, your visual experience is that the world (and not your eye) is moving. You can demonstrate this by covering one eye and then pressing gently on the side of the other; the visual scene appears to move. However, if you cover one eye and then move the other from side to side in the normal fashion (i.e., without the aid of your finger), you will have the normal sensation of vision with a stable visual field. The process of attenuation by the predicted state is not restricted to touch and vision; when we speak, the predicted auditory consequences are relayed to auditory sensory areas where incoming sound is attenuated (Greenlee et al., 2010).

The comparator model of motor control, and in particular the proposal of internal comparator mechanisms, has acquired considerable support (e.g., Kawato and Wolpert, 1998) and there is emerging evidence that such a model can be instantiated within the networks of the brain (Frith, 2005b; Ramnani, 2006; Knolle et al., 2012).

### The comparator account of the sense of abnormal agency for bodily action

The comparator account of the sense of agency for bodily action proposes that as well as explaining the adjustment and control of motor action, the mechanisms of the motor control system can also provide an account of the sense of agency and its disruption in delusions of alien control (Frith et al., 2000a,b; Blakemore et al., 2002; Frith, 2005a, 2012). The account proposes that in normal cognition the generation of the predicted state underlies the sense of self-agency (see Figure 1). Firstly, during ordinary movement, the comparison of the predicted state to the incoming sensory information (in the third comparator, C3) should reveal a match, allowing self-generated movements to be distinguished from sensory feedback that is non self-generated, and giving rise to a *feeling of self-production*. In addition, the account holds that the mere generation of the predicted state may also contribute to the sense of agency, by giving rise to a *feeling of initiation* (Blakemore et al., 2002).

In schizophrenia, the predicted state is proposed to be faulty in some way, interfering with both of these aspects of the sense of agency and giving rise to delusions of alien control. Firstly, a faulty or absent predicted state leads to a lack of the feeling of initiation. Because the patient is not aware of having initiated the movement, “[i]t is as if the movement, although intended, has been initiated by some external force” (Blakemore et al., 2002, p. 240). Secondly, a faulty or absent predicted state would lead to a mismatch in the third comparator, meaning that the sensory consequences of the self-generated movement are not attenuated. It is proposed that this failure of attenuation leads to a feeling of non-self-production.

The account has received considerable empirical support from studies indicating that problems in predicting the sensory consequences of action are associated with schizophrenia (Blakemore et al., 2000; Shergill et al., 2005; Leube et al., 2010), as well as evidence of functional and structural abnormalities in schizophrenia in many of the brain regions suggested to play a role in motor prediction (for recent overviews of this evidence see Farrer and Franck, 2007; Voss et al., 2010; Pynn and DeSouza, 2013). There are also plausible neurobiological accounts consistent with the cognitive account (Fletcher and Frith, 2009; Whitford et al., 2012).

A popular way to classify accounts of the sense of agency has been to draw a distinction between “top-down” and “bottom-up” approaches. Top-down approaches are those that explain misattributions by appealing to disruptions in interpretive processes incorporating conceptual information about the self (e.g., Wegner, 2002; Stephens and Graham, 2003). By contrast, bottom-up approaches are those that explain misattributions of agency by appealing to disruptions in subpersonal, automatic, non-interpretive processes. This widespread distinction between top-down and bottom-up etiological accounts mirrors the recent explication of two distinct functional and representational levels at which the sense of agency can be usefully analyzed (Synofzik et al., 2008a). One is the level of *feeling* of agency, which is argued to represent the non-conceptual, low-level feeling of being the agent of an action, at which level the self can only be implicitly represented. The other is the level of *judgment* of agency, which refers to the interpretive, conceptual judgment of being the agent of an action at the level of the narrative self. One way that these levels have been elucidated has been to appeal to the experience of optical illusions (e.g., Bayne, 2011). The Müller-Lyer illusion consists of two lines of identical length; one of the lines has arrows on either end that point inwards, and the other has arrows that point outwards. Even when we are able to make the conceptual judgment that the two lines are of the same length (for instance, after we have measured them), we continue to have the visual experience of them as different lengths. Something like this distinction is understood to hold for subjective experiences such as the experience of agency for inner speech (Bayne, 2011). A person may reach the conceptual judgment that an episode of inner speech was self-produced (for instance, on the basis that there is no one else in the room), but they may nonetheless have the first-person *feeling* that the episode was non self-produced.

The comparator account of the sense of agency provides a bottom-up account that explains the cognitive generation of subpersonal *feelings* of agency. As such it has been criticized for suggesting that a non-conceptual feeling of non-self agency could fully account for a conceptual judgment of external agency (e.g., Synofzik et al., 2008a). However, it is worth noting that proponents of the comparator account have always maintained that additional disruptions to the patient’s belief system are required to explain how the abnormal feelings of agency are interpreted in an irrational way (Blakemore et al., 2002; Frith, 2012). Most recently, Synofzik et al. have incorporated the comparator account of the sense of agency into their multifactorial weighting model (MWM). This model holds that a *variety* of top-down and bottom-up cues—including feelings of agency issuing from the motor control system—are ordinarily integrated to give rise to the sense of agency (Synofzik et al., 2008a,b, 2009a,b, 2013; Synofzik and Voss, 2010; Synofzik and Vosgerau, 2012).

### The comparator account of the sense of abnormal agency for inner speech

**Figure 2:**
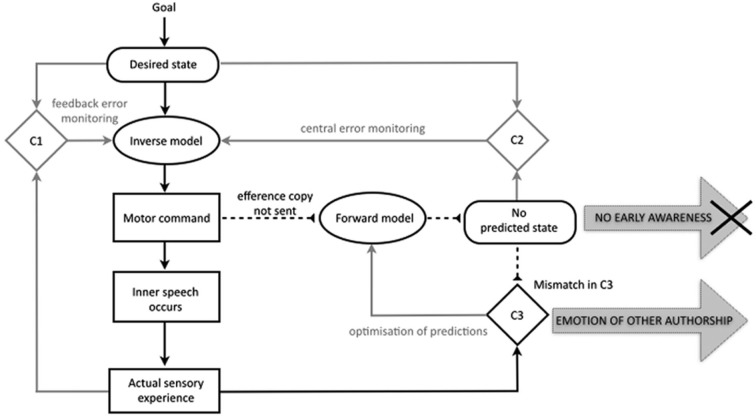
**The traditional comparator account of inner speech in the motor control system and the sense of agency for inner speech in schizophrenia, with relevant processes in black**. The account holds that inner speech is, like overt speech, the direct product of the motor command, and that an efference copy is also produced. In pathology, a failure to send an efference copy of the motor command means that no predicted state is generated, leading to a *lack of early awareness* of the inner speech, and an *emotion of other authorship*. Based on Jones and Fernyhough (2007); with basic features of the motor control system as in Figure 1 (based on Frith et al., 2000b; Blakemore et al., 2002; Synofzik et al., 2008a).

Besides explaining misattributions of bodily actions such as delusions of control, proponents of the comparator model have often aimed to extend the account to explain the misattribution of mental acts (Feinberg, 1978, 2011; Frith, 1992, 2012). In recent years this has taken shape in the proposal that the same motor control-based disruption in predictive processes may impact the experience of inner speech, underlying symptoms of AVH and even thought insertion. Up to one fourth of our conscious mental life is comprised of “talking” to ourselves silently in our minds (Heavey and Hurlburt, 2008). Since AVH consists of reporting a voice when none is present, it is plausible that inner speech may form the basis of the hallucinatory experience. A variety of cognitive models have been proposed to explain how we might ordinarily come to have the subjective, internal experience of thought in natural language (e.g., Levelt, 1983; Kinsbourne, 2000; Fernyhough, 2004; Kosslyn, 2005; Carruthers, 2006; Baddeley, 2007). The comparator account of inner speech holds that similar motor control processes will underpin the production of sentences in natural language whether they are “spoken” internally or externally. On this basis, several theorists have provided detailed accounts of a comparator account for misattributed inner speech (Seal et al., 2004; Jones and Fernyhough, 2007; Langland-Hassan, 2008; Whitford et al., 2012). Jones and Fernyhough (2007) provide the clearest and most comprehensive explication of such an approach. Their account is outlined in Figure 2, showing both the specification of inner speech and the proposed disruptions in schizophrenia.

The basic proposal is that inner speech, like outer speech and other bodily acts, is a product of the motor control system in such a way that it is compared to, and attenuated by, a predicted state. The model holds that in the normal case of inner speech a goal generates a representation of the desired state, and a motor command is issued. The motor command results not only in the occurrence of the action (in this case, inner speech occurs) but also in the generation of the efference copy and predicted state.

Just as with the comparator account of the sense of agency for bodily actions, the approach holds that deficits in the predicted state result in an abnormal experience of agency. The account is a fairly direct transposition of the comparator account as it applies to bodily actions. Firstly, a failure to generate a predicted state results in a lack of feeling of initiation for the inner speech or, to use the term employed by Jones and Fernyhough (2007, p. 395), “no emotion of self-authorship”. Secondly, the same failure to generate a representation of the predicted state results in a mismatch in the third comparator (C3), resulting in the episode of inner speech being classified as *non-self* in origin, or, to use Jones and Fernyhough’s (2007, p. 395) phrase, resulting in an “emotion of other-authorship”. These two factors are posited to combine to create a conscious experience of non-self agency, which is then interpreted by “top-down” factors, i.e., conscious judgments, to give rise to an explicit misattribution of agency.

## Problems with the traditional comparator account of misattributed inner speech

The basic explication of the comparator account applied to inner speech—that inner speech is normally predicted and attenuated and that failures in this process contribute to misattribution—is widely accepted both within the literature on the sense of agency and beyond (e.g., Li et al., 2002; Johns et al., 2006; Vosgerau and Newen, 2007; Synofzik et al., 2008a; Carruthers, 2011; Ford and Mathalon, 2012; Whitford et al., 2012). In contrast to this consensus, we argue that there are fundamental problems with the comparator account as it is currently specified. These problems mean that both the basic model of how inner speech is specified within the motor control system and the account of how deficits in prediction lead to misattributions of agency are untenable. The critique will focus on Jones and Fernyhough’s version of the comparator account, but the main points apply to any version of the account that maintains that inner speech is attenuated by a predicted state.

Before outlining our concerns with the current account, it is important to note that we will not challenge the basic proposal that the motor control system may be involved in the production of inner speech. Firstly, while the comparator model of the motor control system was originally posited to account for motor-to-somatosensory predictions in motor action, there is emerging electrophysiological and behavioral evidence that the extension of this model to motor-to-auditory predictions is plausible (Bäß et al., 2008; Greenlee et al., 2010; Weiss et al., 2011; Knolle et al., 2012). Secondly, there are several strands of evidence indicating that inner speech may be a product of the motor control system (for a review see Stephane et al., 2001). This includes developmental evidence that inner speech is related to early private speech (Berk, 1992), evidence of structural similarities between speech and inner speech (Dell and Repka, 1992), as well as brain imaging data which support the hypothesis that the same mechanisms are involved in both inner and outer speech (Jeannerod, 2006). Moreover, to accept a motor system route to inner speech does not rule out the possibility of alternative routes to verbal imagery, for example, involving the reconstruction of perceptual memories in modality specific cortices (Kosslyn et al., 2001; Kosslyn, 2005; Moulton and Kosslyn, 2009). The critique offered here therefore, relates not to *whether* inner speech is functionally specified in the motor system, but rather* how* it is specified.

### Problems with the specification of inner speech in the traditional comparator account

As described above, the existing comparator account of misattributed inner speech assumes that inner speech holds the same functional position in the motor control system as actual speech. This aspect of the account, and in particular the related proposition that inner speech is compared in the third comparator and attenuated by the predicted state, forms a crucial aspect of the approach’s account of the etiology of AVH. Despite this, Jones and Fernyhough provide no clarification of the notion of inner speech they have in mind, nor of its cognitive specification. One clue comes from their diagrammatic representation of the model, which indicates that the occurrence of inner speech based on the motor command results in an “actual sensory experience” (see Figure 2). Despite this nomenclature, there is strong reason to believe that Jones and Fernyhough do *not* mean that the production of inner speech results in external sensory output (such as low level vocalization or muscle movements). Not only do they clearly refer to inner speech as “purely cognitive” throughout the article, they also expend considerable effort constructing an argument (drawing on a Vygotskian (Vygotsky, 1934/1987) developmental notion of private speech) for why we should expect a purely cognitive event such as inner speech to be the product of the motor control system in the first place. If they meant the inner speech output in their model to consist of low level vocalization with actual sensory consequences, then such arguments would not be required. If the notion of inner speech they have in mind is purely cognitive, then the output which they have labeled as “actual sensory consequence” would be better described as imagery (quasi-perceptual representation) of what the actual sensory consequences might have been had the speech been produced. Such a characterization would be in line with both their own description of inner speech as “purely cognitive” and with standard cognitive characterizations of inner speech (e.g., Carruthers, 2006; for an overview see Vicente and Martinez-Manrique, 2011).

Given the information provided by Jones and Fernyhough, this explication seems the most plausible way to characterize the notion of inner speech in their model. However, further questions remain. We leave open the question of the modality of inner speech in their account. It is likely that they would follow other theorists (e.g., Tian and Poeppel, 2012) in positing that this quasi-sensory representation could occur in either the auditory or the motoric and kinesthetic modalities, or in all three. More crucially, it is unclear under their account what mechanisms are supposed to generate the quasi-perceptual representation of inner speech. In the case of overt speech as specified in the original model of motor control, the motor command causes the bodily movement to occur, actual sensory consequences follow, and these are picked up by the sensory system and compiled into a representation (the “estimated actual sensory consequences”, see Figure 1). In Jones and Fernyhough’s model, the process by which the motor command leads to quasi-sensory representation of inner speech remains unspecified.

Moreover, it is unclear why there is a need to propose any new mechanisms for the generation of inner speech. If inner speech consists of a quasi-sensory representation of the likely consequences of a given act of speech, then the motor control system as originally specified already contains such a representation. Recall that, according to the original comparator model of the motor control system, an efference copy of the motor command is sent to the forward model, which generates representation of the predicted sensory consequences of performing the motor command. We know that this predicted state must be in the same representational format as the posited inner speech of Jones and Fernyhough, since, according to them, both are inputs to the third comparator. If the efference copy and forward model already issues a quasi-perceptual representation of the predicted sensory consequences of performing a given speech act, then would it not be more parsimonious to consider that *this* representation—the predicted state—would form the basis for inner speech? In the normal case of overt action, the predicted state is a subpersonal representation, but all that would be required to generate the conscious experience of inner speech would be to suppress the motor command and make the predicted state available to consciousness. Moreover, this specification of inner speech as derived from the predicted state is just as consistent with the motivations provided by the Vygostkian development view of language that Jones and Fernyhough offer. Firstly, inner speech is still a product of the motor control system. Secondly, the predicted state is compared to the desired state in the second comparator, providing a mechanism by which inner speech could be monitored and corrected.

In light of these issues, this traditional version of the comparator account of the sense of agency for inner speech faces several challenges. Either the notion of inner speech needs to be elaborated in order to explain how it is functionally different from the predicted state, or, if it is *not* different, there needs to be an explanation of why the motor control system would generate the same state twice, and what would be gained from comparing it to itself.

The alternative proposal we have offered—that the predicted state could form the basis for inner speech—is not only more parsimonious and well-defined than that provided by Jones and Fernyhough, but also more consistent with leading theorizing on motor imagery. As part of an extensive research program over a number of years, Marc Jeannerod (2006) has proposed an account of motor imagery based on the workings of the motor control system. According to this influential theory, motor imagery—conscious quasi-perceptual representation of motor acts—is derived from the predicted state. His account forms the basis for the specification of inner speech in at least one leading theory of the architecture of the mind (Carruthers, 2006). Recently, Tian and Poeppel (2012) have expanded on this approach to provide clear specification of how the forward model and predicted state of the motor control system could generate both the sensorimotor and auditory imagery associated with inner speech.

Our analysis of the traditional comparator account of misattributed inner speech suggests that the proposed specification of inner speech in the motor control system is problematic. This in turn calls into question the viability of the current comparator account as a model for misattributions of agency. If inner speech is, as we alternatively propose, derived from the predicted state, then it is not normally attenuated and a mismatch in the third comparator cannot account for its misattribution. It is possible that these problems with the traditional comparator account are at least implicitly recognized by some theorists. After initial enthusiasm in earlier versions of his account (Frith, 1992), Frith himself has become increasingly cautious about applying the comparator account to inner speech (Frith, 2012).

### Problems with the evidence for the traditional comparator account of inner speech

Over the last 15 years a series of studies using electrophysiological techniques has probed the responsiveness of the brain to auditory probes during self-generated speech and inner speech. A primary aim of this research was to test the predictions of the comparator account as it applied to misattributed inner speech. Overall, the results have been interpreted as suggesting that inner speech is normally attenuated, and that there is a failure of attenuation of inner speech in patients with a diagnosis of schizophrenia (Ford et al., 2001a,b,c, 2007; Ford and Mathalon, 2004, 2005, 2012; Heinks-Maldonado et al., 2007). As such, the results appear to provide key evidence in favor of the current comparator account of misattributed inner speech.

In this section we closely examine the details of these studies and argue that there are problems with this common interpretation of the data. We suggest that even if we were to leave aside the analysis provided in the previous section and accept for the sake of argument that inner speech could plausibly be specified as functionally equivalent to overt speech in the motor control system, the data from these electrophysiological studies cannot be taken as supporting the traditional comparator account of inner speech.

The auditory N1 is a negative-going event related potential (ERP) generated in the auditory cortex by transient auditory stimuli, and has been the primary dependent measure on which the series of studies by Ford et al. have been based. It reaches its peak approximately 100 ms after stimulus onset and is measured by electroencephalography (EEG). Magnetoencephalographic (MEG) studies measuring the N1’s magnetic counterpart, the N1m, have shown that, while a subject is talking, responsiveness of the auditory cortex to 1000 Hz tone probes is dampened and delayed compared to while a subject is reading silently (Numminen et al., 1999), or simply listening (Curio et al., 2000). In line with the comparator account of motor control, the reduction of N1m during talking in these studies was attributed to the dampening effect of the predicted state. These findings are consistent with a large body of research demonstrating the attenuation of sensory consequences during bodily action across modalities and across the animal kingdom (Crapse and Sommer, 2008).

Ford et al. expanded on this research to investigate N1 responsiveness to auditory stimuli not only in healthy controls, but also in patients with a diagnosis of schizophrenia. The majority of their studies focused on the differences in N1 responsiveness to auditory probes during overt talking as compared to a baseline condition during which subjects heard the auditory probes and were asked simply to focus on a fixation point. Ford et al. describe this as the Talk/Listen paradigm. In some studies the talking itself provided the auditory probe (and was played back during the listen condition). In other studies separate auditory probes were used (e.g., speech sounds [/ba/] and noises [broadband]).

Across these studies involving overt speech, healthy controls showed a significant difference in N1 responsiveness between the baseline and talking conditions, with N1 responsiveness to the auditory probe dampened while talking. In line with the comparator account of the motor control system and the previous research described above, these findings were interpreted as indicating that the predicted state attenuates incoming sensory information during speech.

By contrast, the patient group showed no such difference in N1 responsiveness between the talking and baseline conditions. This was taken to indicate a failure of attenuation of incoming sensory information, as predicted by the comparator account of misattributions of bodily agency in schizophrenia. While these results do provide good evidence of attenuation (and failure of attenuation) during overt speech, it is not straightforward to assume that they can be extrapolated to shed light on covert actions like inner speech. As noted in the previous section, it is problematic to presume that inner speech plays the same functional role in the motor control system as overt speech, and there is evidence that an alternative model of inner speech in the motor control may be appropriate. In the present context, to draw conclusions about the posited attenuation of inner speech from data relating to overt speech is to beg the question.

Given this, only evidence that inner speech is *itself* attenuated, and that failures of this attenuation are connected to schizophrenia, can be directly taken as evidence of the current comparator account of misattributed inner speech. Just one of the studies conducted by Ford et al. investigated levels of N1 responsiveness during inner speech (Ford et al., 2001a; Ford and Mathalon, 2004). Drawing on the type of comparator account of inner speech suggested by Jones and Fernyhough, the authors predict that engaging in inner speech will lead to a reduction in N1 responsiveness due to attenuation by the predicted state associated with the production of inner speech. As with the studies involving overt speech, during a baseline condition subjects simply focused on a fixation point and listened to the auditory stimuli. In the inner speech condition the participants engaged in what the authors refer to as “directed” inner speech by silently repeating statements (e.g., “That was really stupid”). In both the inner speech and baseline conditions, auditory probes were presented (e.g., speech sounds [/ba/], noises [broadband]) and N1 response to these stimuli was recorded.

The key results from the inner speech study were broadly similar to those from the studies involving overt speech. Firstly, in healthy controls the N1 responsiveness to the auditory probes was reduced in the inner speech condition as compared to the baseline condition. The authors take it that the production of inner speech in the motor control system in this condition has given rise to a predicted state, which has in turn attenuated not only the inner speech itself, but also N1 responsiveness. Thus, the result that N1 responsiveness was reduced during the inner speech condition in the control subjects has been taken to support to the basic proposition that inner speech is normally attenuated, as proposed in the traditional comparator account of misattributed inner speech.

In addition, the subjects with a diagnosis of schizophrenia demonstrated *no* difference in N1 responsiveness to the auditory probes between the inner speech and baseline condition. Ford et al. (2001a) suggest this may be because in the case of the inner speech produced by patients the predicted state “was not functioning properly” and so “auditory cortical responsiveness… might not have been dampened” (p. 1915). In line with the traditional comparator account of misattributed inner speech, this failure of attenuation of the N1 response is posited to reflect a failure of attenuation of inner speech itself, which contributes to symptoms such as AVH and thought insertion by causing a failure of the “self/other signal” (p. 1915) or as they put in a later description (Ford and Mathalon, 2004), leading to “the misperception that […] thoughts have an external source” (p. 43).

These interpretations of the key data as being supportive of the current comparator account of misattributed inner speech have been repeated by Ford et al. in several reviews of the original study (e.g., Ford and Mathalon, 2012) and in turn referenced across the literature on the sense of agency for thought (e.g., Langland-Hassan, 2008). However, it is clear from a closer reading of Ford et al.’ broader research program that this is not the only, or even the best, interpretation of the key data from the inner speech study. Firstly, it is important to note that the study does not directly measure attenuation of inner speech, but rather draws inferences about attenuation of inner speech from levels of N1 responsiveness. For this reason, interpretation of the N1 responsiveness data requires an *a priori* assumption about the posited nature and direction of the relationship between N1 responsiveness and any supposed inner speech attenuation.

Specifically, the interpretation described above rests on the assumption that a reduction in N1 responsiveness is the result of attenuation by the predicted state and can be taken as a direct indication that inner speech is itself also being attenuated. To put it another way, the interpretation rests on the assumption that a reduction in N1 responsiveness reflects a properly functioning predicted state and properly attenuated inner speech. Elsewhere in discussing the same series of studies, however, the authors make the opposite *a priori* assumption, positing that reduced N1 responsiveness could reflect a *failure* of predicted state, and as indicating that inner speech itself has *not* been attenuated (see details below). This is extremely problematic; if reduced N1 responsiveness can be plausibly interpreted as reflecting either a properly functioning predicted state or a failure of the predicted state, then it is impossible to draw firm conclusions about either the specification of inner speech (whether it is normally attenuated) or the role of prediction failure in schizophrenia from the N1 data gathered in these studies.

Given the seriousness of this problem, it is worth spelling out in detail this alternative contradictory framework as posited by the authors. Firstly, Ford et al. make clear that attenuation by the predicted state is not the only mechanism by which the dependent measure of N1 responsiveness may be reduced. Acoustic interference (for instance, listening to speech) is another possible mechanism for reduction of N1 responsiveness to auditory probes, because the auditory cortex is already engaged (Ford et al., 2001b; Ford and Mathalon, 2004). For instance, Ford et al. appeal to this mechanism to explain why, in a third listening condition, N1 responsiveness is at its lowest as compared to both baseline and speech conditions for both patients and controls, even though attenuation is clearly not at work (since the participants are not engaging in speech); the reduction is argued to be the result of acoustic interference from the short bursts of heard speech (Ford et al., 2001b, p. 547).

Importantly, they appeal to this process of acoustic interference again when explaining a set of findings from the baseline conditions within studies. In this case their appeal to acoustic interference has important implications for their interpretation of the relationship between inner speech, the predicted state and N1 responsiveness. Recall that in the baseline condition, individuals simply sit and listen to auditory probes. Across the various studies involving both overt and covert speech the level of baseline N1 responsiveness to auditory probes was lower in the patient groups than in the control group; that is, in the baseline condition the patient’s N1 responsiveness seemed to have been dampened as compared to baseline responsiveness of control subjects. Ford et al. explain this finding by appealing to differential levels of acoustic interference from inner speech in the control and patient groups. This differential level comes not from different *amounts* of inner speech—as they say, it is “likely that both control subjects and patients engage in internal dialogue”, during the baseline condition (Ford et al., 2001b, p. 547)—but rather from differences in the level of *attenuation* of the inner speech between the control and patient groups. Specifically, they posit that inner speech in the patient group is not attenuated (due to a failure of the predicted state, as proposed by the comparator account), meaning that it causes greater acoustic interference, thereby reducing N1 responsiveness. In the control group they posit that inner speech is correctly attenuated (in line with the comparator account) and therefore interferes with the N1 less, meaning N1 responsiveness is not reduced.

This explanation of the likely relationship between the attenuation of inner speech and N1 responsiveness offered in interpreting the data between the baseline conditions is in direct contradiction of the interpretation offered in relation to the key findings discussed above. In the key findings above Ford et al. interpret reduced N1 responsiveness (in the control group as compared to the patients in the inner speech condition) as reflecting *functioning* attenuation of inner speech; when inner speech is correctly attenuated by the predicted state, the N1 responsiveness is attenuated in the same way. But in discussing the baseline findings, Ford et al. posit the inverse relationship, whereby reduced N1 responsiveness (in the patient group as compared to the control group) reflects a *failure* of attenuation of inner speech; unattenuated inner speech interferes with the auditory cortex, reducing N1 responsiveness.

That these two proposals about the relationship between inner speech, attenuation by the predicted state and N1 responsiveness are both available is not in itself problematic; both are theoretically driven and internally consistent. What is problematic is the coexistence of them in interpretation of the same set of data without making their contradictions explicit. More simply, it is impossible to draw any conclusions about the compatibility of the key N1 responsiveness results with the comparator account of misattributed inner speech if reduced N1 responsiveness could plausibly indicate both *functioning* attenuation or a *failure* of attenuation of inner speech. Had the findings revealed the opposite pattern of findings for the key comparison between control and patients in the inner speech condition—i.e., had they found that N1 response was reduced in the patients rather than the controls—this too could have been deemed in keeping with the traditional comparator account of misattributed inner speech, simply by appealing to the alternative *a priori* assumption regarding the relationship between N1 responsiveness and inner speech attenuation.

The above analysis reveals one additional note of caution about interpreting the results from the inner speech study. It is clear that Ford et al. did not control for the possibility that subjects would engage in spontaneous inner speech during the baseline condition. In fact, as noted above, in discussing the differences between the baseline conditions in a similar study, Ford et al. *assume* that participants were engaging in inner speech during the baseline. This means that there are at least two alternative explanations for the key findings from the inner speech study. Firstly, the difference in patterns of N1 responsiveness could simply be due to differential levels of spontaneous inner speech in the baseline condition. Suppose, for instance, that patients tended to engage in spontaneous inner speech in the baseline condition while those in the control condition did not; the additional acoustic interference provided by inner speech in the control subjects would explain the reduction in N1 responsiveness in the inner speech as compared to the baseline condition, while the lack of difference between the two conditions in the patient groups would be attributable to the fact that N1 response was already affected by acoustic interference from inner speech in the baseline condition. Alternatively, it could be that levels of spontaneous inner speech were the same in both patients and controls, but that the level of attention differed between groups; if patients tended to pay more attention to their spontaneous inner speech the same pattern of key results would be expected. Notably, neither of these plausible explanations for the pattern of data from the inner speech study makes any appeal to mechanisms by which inner speech is predicted or attenuated, as posited by the traditional comparator account of misattributed inner speech.

The above analysis calls into question the leading evidence for the current comparator account of misattributed inner speech. As pointed out by Langland-Hassan (2008), there are other research programs employing brain imaging which demonstrate results *consistent* with the comparator account, but those other studies—showing, for example, that the nervous system in patients with AVH behaves as it would during normal speech perception (Dierks et al., 1999)—are also consistent with alternative models of AVH which do not appeal to a model of attenuated inner speech (e.g., Allen et al., 2008). To date, the results from across the Ford et al. studies have been held up as the leading evidence in favor of the current model. This analysis reveals that the common interpretation of these key data from the inner speech study as supporting the comparator account of misattributed inner speech is problematic. Not only is it impossible to conclude from these data that the attenuation of inner speech is faulty in schizophrenia, but it is also impossible to conclude that inner speech is normally attenuated.

## A new comparator account of misattributed inner speech

Given the emerging evidence that motor prediction failures are associated with symptoms of hallucination and delusion in schizophrenia (Frith, 2012), and the plausibility of the comparator account as it applies to bodily action, there is a strong motivation to seek a motor control based account of symptoms such as AVH. However, in the previous two sections we have pointed out fundamental flaws in the traditional comparator account of misattributed inner speech, and highlighted inconsistencies in the interpretation of the electrophysiological data commonly taken to support the account.

There is one existing alternative comparator account of the sense of agency for inner speech that (seemingly inadvertently) sidesteps these problems by re-conceptualizing the process of prediction in the motor control system as a process of filtering. Langland-Hassan (2008) suggests that the idea of prediction proposed by Miall et al. (1993) and Wolpert and Ghahramani (2000) is not the only way in which Sperry (1950) and Holst and Mittelstadt (1950) original model of the motor control system could be cashed out, arguing that in the case of visual and auditory modalities the motor control system could calculate the needed cancellation of the incoming sensory information without ever generating a full prediction of the actual input. Crucially, then, his filter model “does not require that the ‘predictive’ signal itself be a quasi-visual state” (Langland-Hassan, 2008, p. 383). This account may avoid some of the problems of the traditional comparator account by avoiding a proposal of “double prediction”, but it does not provide a plausible alternative. Contemporary theory and research indicates that the idea of predictive forward models based on efference copies is ubiquitous across sensory domains (Pynn and DeSouza, 2013). Not only does Langland-Hassan’s account stand counter to this evidence, but it also entails a rather puzzling and unjustified split in the functioning of the motor control system’s forward modeling. While he proposes that prediction does not occur in the visual and auditory modalities, in the case of the somatosensory and kinesthetic domains Langland-Hassan holds that the conceptualization of forward modeling as a process of prediction is valid (Langland-Hassan, 2008, p. 381).

In this section we outline a new comparator account that we take to provide the most viable model of how prediction failures in the motor control system could give rise to misattributions of inner speech. Unlike the traditional comparator account it involves a clear and cognitively justified specification of inner speech, is in line with leading theories of motor imagery, and does not entail duplication of states in the motor control system. Unlike Langland-Hassan’s account it does require a radical re-conceptualization of the forward-modeling processes of the motor control system.

The new account is based on a model of inner speech production derived from the motor imagery literature (Carruthers, 2006; Jeannerod, 2006; Tian and Poeppel, 2012) and is consistent with recent arguments that mental imagery is likely to be based on full-blown simulation (Moulton and Kosslyn, 2009). This model of ordinary inner speech assumes that inner speech is directly derived from the predicted state. Inner speech begins, like overt speech, in the formation of an intention (which can be a motor intention, see Pacherie, 2008), leading to the generation of the desired state and the required motor command. As in the case of actual speech, an efference copy of the motor command is sent to the forward model and a prediction of the sensory consequences of the given speech act is produced.

It is clear from recent research that the predicted state can comprise representations across sensory modalities, including somatosensory, visual and auditory (Cullen, 2004; Pynn and DeSouza, 2013). In line with the recent model of inner speech proposed by Tian and Poeppel (2012), the model outlined here holds that the prediction that forms the basis of inner speech can incorporate both somatosensory (articulation imagery) and auditory (hearing imagery) modalities (see also Tian and Poeppel, 2010). The predicted state, which during overt speech normally remains in subpersonal processing, is made available to higher levels of processing (for example, via global broadcasting, see Carruthers, 2006). This process results in the first-person conscious experience of the episode of inner speech. The proposal that the predicted state may form the basis of the sensory content of inner speech is supported by recent behavioral studies (Scott, 2013; Scott et al., 2013).

In contrast to overt speech, in the case of inner speech the motor command is suppressed. Because the motor command is suppressed, there are no actual sensory consequences and there is no comparison in the third comparator. However, in line with similar models of motor imagery (Grush, 2004; Jeannerod, 2006), and specifically inner speech (Tian and Poeppel, 2012), the present model holds that the comparison in the second comparator between the desired and predicted states still occurs. We propose that during ordinary inner speech the match between the desired state and the predicted state in the second comparator contributes to the sense of agency for inner speech. The idea that the comparison in the second comparator might contribute to the sense of agency is not new. In at least one discussion of the comparator account of the sense of agency for bodily action, Frith has indicated that as well as the sense of initiation (derived from the mere production of the predicted state) and the sense of self-production (derived from the match in the third comparator) the match between the intended and predicted state may evoke a sense of “being in control” (Frith, 2005b, see also Synofzik et al., 2008a, p. 221).

**Figure 3:**
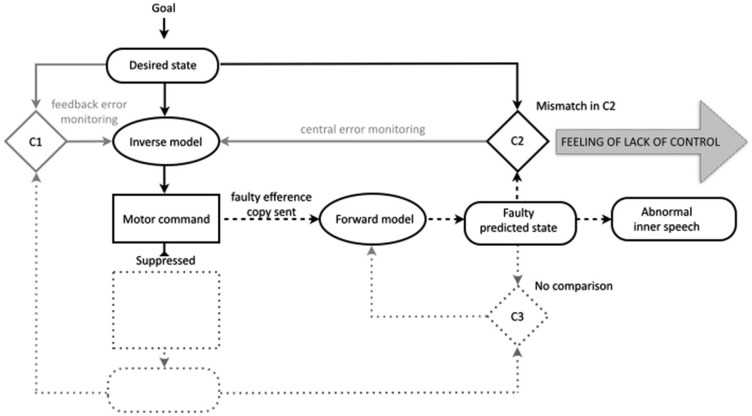
**The new comparator account of inner speech in the motor control system, also showing proposed disruptions to the experience of inner speech in schizophrenia**. The efference copy allows the production of the predicted state to form the basis for conscious mental imagery (inner speech), while the motor command is suppressed (those aspects of the motor control system therefore not implicated in inner speech are shown dotted). In ordinary inner speech the match in comparator two between the predicted and desired states gives rise to a feeling of control. In schizophrenia, failures in the predicted state directly affect the conscious experience of the resultant inner speech (abnormal inner speech)* and* lead to a mismatch in the second comparator, leading to abnormalities in the feeling of intentional control.

With this framework in place it is possible to provide a unified and plausible account of how failures in motor prediction could contribute to the misattribution of inner speech (Figure 3). Following the traditional comparator account, we propose that in schizophrenia there are disruptions somewhere in the process of efference copy production and forward modeling, leading to a faulty or inaccurate predicted state. We leave unspecified the precise nature of this fault. The faulty prediction is proposed to potentially occur across the various modalities that contribute to ordinary inner speech (e.g., auditory and somatosensory). Thus, the errors in prediction could encompass incorrect specification in one modality (i.e., predicting the speech as louder or quieter, faster or slower), or incorrect specification across modalities (i.e., predicting the speech as composed of more or less auditory imagery relative to motor imagery). In line with traditional versions of the comparator account the deficit in the predicted state is also proposed to be sporadic, meaning that the predicted state will be accurate most or some of the time. Finally, these sporadic errors in the predicted state will lead to instances of a mismatch in the second comparator, whereby the predicted state will no longer match the desired (intended) state.

The proposed deficit in the predicted state is likely to have at least two distinct consequences for the phenomenology of the associated inner speech. The first is that the prediction error would directly translate into the patient’s conscious experience of the resultant inner speech. In comparison to their ordinary inner speech, the individual could find that they experience inner speech which is unusual across any of the dimensions associated with the prediction of the sensory consequences of speech; the inner speech could be unusually slow/fast, unusually loud/quiet, unusually auditory in nature, unusually clear/unintelligible etc. It is these characteristics, which would differ from the characteristics of ordinary, correctly predicted inner speech, that are proposed lead the inner speech to be experienced as another person’s voice. It is possible that the precise nature of the prediction errors would vary between, and even within, individuals, meaning that the proposed deficit could give rise to a wide variety of phenomenologically unusual cases of inner speech. Secondly, the mismatch in the second comparator between the desired state and the predicted state would mean that an unusual feeling of agency would accompany the associated inner speech, potentially a feeling that the inner speech is outside of intentional control. The sporadic nature of the deficit means that these experiences would be interspersed with episodes of phenomenologically ordinary inner speech accompanied by an ordinary feeling of agency.

### The new comparator account and evidence from the phenomenology of AVH

A primary motivation for developing a comparator account of misattributed inner speech is to provide an etiological account of AVH in schizophrenia. The new account that we have proposed fits well with emerging evidence on the phenomenology of voice-hearing in schizophrenia. The account predicts that AVH would be experienced as outside of intentional control and unusual across a range of phenomenological dimensions related to sensory prediction. These predictions are in line with standard characterizations of AVH which hold that, along with a phenomenology of “externality”, AVH are commonly experienced as both uncontrolled and compellingly perceptually real (Moritz and Larøi, 2008; Waters et al., 2012; Wu, 2012).

The account’s predictions are also in line with a recent study which confirmed that AVH differ from patients’ ordinary inner speech along a number of dimensions related to their perceptual phenomenology, including their speed (compared to a normal rate of speaker), intelligibility (understandable or garbled) and volume (Langdon et al., 2009). Moreover, while patients were able to describe the nature of various vocal characteristics of their AVH (the perceived gender, age, accent and class of the voices), the majority reported that their ordinary inner speech was free of such characteristics and “more like words in the head than a voice in the head” (Langdon et al., 2009, p. 661). As several theorists have concluded, the traditional comparator account of misattributed inner speech struggles to explain these phenomenological differences, since it predicts only differences in the experience of agency (Langdon et al., 2009; Wu, 2012).

In addition, our new account proposes that the precise effect of prediction failure could differ between individuals, and would therefore predict that AVH could vary across individuals in terms of any phenomenological dimension associated with prediction, including spatial location (predicting how close the voice will sound), identity of the voice (predicting the tone and timbre of speech), and reality (prediction of auditory characteristics in general). This is in line with the emerging evidence that voice-hearing in schizophrenia is a diverse and heterogeneous experience which varies along a number of phenomenological dimensions, including those commonly held to characterize the experience (Junginger and Frame, 1985; Chadwick and Birchwood, 1994; Oulis et al., 1995; Nayani and David, 1996; Leudar et al., 1997; Watkins, 1998; Stephane et al., 2003; Jones, 2008; Moritz and Larøi, 2008; Daalman et al., 2011; McCarthy-Jones and Fernyhough, 2011). For example, Stephane et al. (2003) found variation in terms of the clarity of AVH (ranging from clear, like external speech, to deep, like thinking in words), personification (e.g., whether it was a male or female voice), loudness (from not having loudness at all, to being softer than or as loud as normal speech), whether voices outside were within or outside of normal hearing range, and whether the voice was attributed to themselves or to another agent. The traditional comparator account of misattributed inner speech struggles to explain this variation between individuals.

### Open questions and future research

The new comparator account of misattributed inner speech draws on a significant reconceptualization of inner speech in the motor control system and makes novel predictions about the likely consequences of motor control failure, thus prompting new research questions and reshaping existing ones. The new model should be of particular interest to researchers investigating the neurocognitive basis of misattributions of both speech and inner speech within the comparator account framework. We have highlighted ambiguities in the way in which Ford et al. have interpreted their findings on the electrophysiological basis of inner speech, appealing both to the traditional view that inner speech is attenuated and an alternative view in which it is inner speech does the attenuating (see Section Problems with the Evidence for the Traditional Comparator Account of Inner Speech). It is hoped that the explication of a new comparator account may provide a clearer framework in which to interpret data from these and future studies.

Another question relates to the viability of the theoretical account of inner speech on which the account is based (e.g., Tian and Poeppel, 2012). Questions remain about how such a theoretical model of inner speech would be instantiated within the networks of the brain (for an overview of possible neural instantiation of the basic comparator account of motor control, see Ramnani, 2006), and how it relates to other models of verbal thought, inner speech and auditory imagery (e.g., Levelt, 1983; Kinsbourne, 2000; Fernyhough, 2004; Kosslyn, 2005; Kraemer et al., 2005; Carruthers, 2006; Baddeley, 2007; Leaver et al., 2009). The model of inner speech also faces phenomenological questions. If ordinary inner speech is derived from the predicted sensory consequences of a motor command to speak, why, for many individuals, is inner speech ordinarily experienced as silent (e.g., Langdon et al., 2009)? And while the new comparator account fits well with the phenomenology of voice-hearing in schizophrenia, there are some elements of the phenomenological data that it struggles to explain, such as apparent differences in the form, pragmatics and content of patients’ inner speech and AVHs (Langdon et al., 2009). There are two possible approaches to making our new account compatible with this type of evidence. The first would be to appeal to a higher order conceptual process that interacts with the outputs of the motor control system such that it is only the combination of the two processes that leads to the experience of AVH. Under this picture, only inner speech that is both the product of faulty prediction *and* has a certain type of content (for example) would be experienced as a voice. This approach would be similar to Synofzik et al.’s multifactorial weighting model, which holds that a variety of top-down and bottom-up cues are integrated to give rise to the experience of agency (Synofzik et al., 2008a,b, 2009a,b, 2013; Synofzik and Voss, 2010; Synofzik and Vosgerau, 2012). Another approach would be to hold that top-down conceptual processes taking into account things like inner speech content and pragmatics could directly impact subpersonal processes, such that prediction errors would be more likely to occur in relation to certain episodes of inner speech.

There are also questions relating to the potential explanatory scope of the new account. In the present article we have focused on the account’s ability to provide an etiological account of AVH, but it is possible that it might be extended to explain delusions of thought insertion or even other thought interference delusions such as thought influence or thought broadcasting. It is difficult to assess the extent to which the new comparator model can provide an explanation for delusions of thought interference because of the paucity of research into the phenomenology of these experiences. Based on the limited evidence currently available, we have previously argued that the phenomenology of thought insertion is best characterized in terms of an anomalous sense of agency for thought, meaning that the new comparator account may provide an account of these delusions (Sousa and Swiney, 2013). However, we argued more specifically that thought insertion is characterized by the sense that a thought as been *generated* or *produced* by another agent, rather than a sense of external intentional control (what we called intentional guidance, Sousa and Swiney, 2013). This more precise characterization is somewhat out of step with the predictions of the new comparator account proposed here. It also remains an open question as to whether inserted thoughts are experienced as perceptually unusual, as the new comparator account would predict.

A related question concerns the modal range of conscious mental imagery that might be affected by the disruptions proposed in the new account. The discussion so far has concentrated on how failures in the prediction of speech acts could give rise to anomalous inner speech, but there is reason to suspect that the account might extend to other types of imagery. Jeannerod (2006) detailed account of motor imagery entails that the full range of imagery (visual, kinesthetic, tactile) is derived from the predicted state of the motor control system, and there is evidence that conscious motor imagery is impaired or altered in schizophrenia across a variety of modalities. Recent research indicates that in schizophrenia imagined movements to grasp a target object show no reliable relationship to target size, suggesting an impairment in imagined movement (Danckert et al., 2002). Another study found that in contrast to patients without symptoms such as delusions of alien control and thought insertion, patients with such symptoms had slowed imagined pointing movements (Maruff et al., 2003). Finally, recent research has revealed that patients with schizophrenia were slower in imagining walking movements as compared to normal controls (Lallart et al., 2012). The researchers undertaking these studies have operated under a theoretical framework in which motor imagery is assumed to derive from the predicted state (as depicted in relation to inner speech in Figure 3). As such, the findings have been taken as providing support for the comparator account of misattributed bodily action, since they indicate problems with motor prediction. But considered in light of the model proposed here, they suggest that prediction failures may have direct consequences for phenomenology across a range of imagistic modalities. If this were the case, the explanatory scope of the account could be widened. For example, some cases of thought insertion appear to refer to “inserted” episodes of visual imagery (Mellor, 1970, p. 17). Also, if failures in the prediction of speech imagery contribute to the hallucination of voices, it is possible that other types of hallucinatory experiences could be explained by appeal to faults in the predictive processes underpinning other modalities of conscious motor imagery.

Finally, it is clear that even if a comparator account of misattributed inner speech is viable, disruptions to the predicted state will not be the only factor that contributes to pathological symptoms. As alluded to in previous versions of the comparator account and spelled out in a recent elaboration of the account (Synofzik et al., 2008a), subpersonal cues from the motor control system are likely to be only one cue contributing to the sense of agency for thought. The comparator account outlined here is intended only to provide a viable picture of how motor control prediction failures could conceivably contribute to misattributions; it is not intended as a full account of the sense of agency for mental acts.

## Conclusions

Since its inception over 25 years ago the comparator account has come to dominate and define the expanding literature on the sense of agency, capturing the imagination of theorists from across the cognitive sciences. Its popularity stems in large part from its potential to provide a unified account of how failures in motor prediction could contribute to the etiology of both delusions of alien control and AVH in schizophrenia. In the case of AVH the comparator account has traditionally assumed that inner speech is cognitively specified in the motor control system in the same way as overt bodily actions, subject to the same processes of prediction and attenuation.

In the present paper we have challenged this traditional account, outlining problems with the specification of inner speech on which it is based and with the interpretation of the electrophysiological evidence commonly cited in its favor. We have provided a new comparator account of misattributed inner speech, appealing to the same failures in motor prediction, but relying on a different specification of inner speech within the motor control system. The new account makes novel predictions about the experience of misattributed inner speech that fit well with the phenomenological evidence on voice-hearing in schizophrenia. It also provides a framework for future neurocognitive research on the effect of motor prediction failures on inner speech.

## Conflict of interest statement

The authors declare that the research was conducted in the absence of any commercial or financial relationships that could be construed as a potential conflict of interest.
